# *miR-103-3p* Regulates the Proliferation and Differentiation of C2C12 Myoblasts by Targeting BTG2

**DOI:** 10.3390/ijms242015318

**Published:** 2023-10-18

**Authors:** Yulin He, Peiyu Yang, Tiantian Yuan, Lin Zhang, Gongshe Yang, Jianjun Jin, Taiyong Yu

**Affiliations:** 1 Laboratory of Animal Fat Deposition and Muscle Development, College of Animal Science and Technology, Northwest A&F University, Yangling, Xianyang 712100, China; heyulin@nwafu.edu.cn (Y.H.); yangpeiyu328@nwafu.edu.cn (P.Y.); yuantiantian@nwafu.edu.cn (T.Y.); zhanglinzl@nwafu.edu.cn (L.Z.); gsyang999@hotmail.com (G.Y.); 2 Key Laboratory of Animal Genetics, Breeding and Reproduction of Shaanxi Province, College of Animal Science and Technology, Northwest A&F University, Yangling, Xianyang 712100, China

**Keywords:** *miR-103-3p*, myoblasts, proliferation, differentiation, *BTG2*

## Abstract

Skeletal muscle, a vital and intricate organ, plays a pivotal role in maintaining overall body metabolism, facilitating movement, and supporting normal daily activities. An accumulating body of evidence suggests that microRNA (miRNA) holds a crucial role in orchestrating skeletal muscle growth. Therefore, the primary aim of this study was to investigate the influence of *miR-103-3p* on myogenesis. In our study, the overexpression of *miR-103-3p* was found to stimulate proliferation while suppressing differentiation in C2C12 myoblasts. Conversely, the inhibition of *miR-103-3p* expression yielded contrasting effects. Through bioinformatics analysis, potential binding sites of *miR-103-3p* with the 3’UTR region of BTG anti-proliferative factor 2 (*BTG2*) were predicted. Subsequently, dual luciferase assays conclusively demonstrated *BTG2* as the direct target gene of *miR-103-3p*. Further investigation into the role of *BTG2* in C2C12 myoblasts unveiled that its overexpression impeded proliferation and encouraged differentiation in these cells. Notably, co-transfection experiments showcased that the overexpression of *BTG2* could counteract the effects induced by *miR-103-3p*. In summary, our findings elucidate that *miR-103-3p* promotes proliferation while inhibiting differentiation in C2C12 myoblasts by targeting *BTG2*.

## 1. Introduction

Skeletal muscle is the largest motor and metabolic organ in the body, and it is also one of the most important components of the body [1]. Skeletal muscle generation involves three primary stages: myogenic progenitor cells undergo proliferation and differentiation into myoblasts, which subsequently differentiate and merge to form myotubes. Finally, myotubes undergo further differentiation to become mature muscle fibers [2]. This intricate developmental process is regulated by a variety of factors, including non-coding RNAs [3], transcription factors [4], and epigenetic modifications [5]. Among these factors miRNAs have emerged as key players, particularly in the regulation of skeletal muscle growth, regeneration, aging, and muscle atrophy [6].

miRNAs, highly conserved non-coding RNAs typically around 22 nucleotides in length, are widely distributed across plants and animals [7]. They bind to the 3’UTR sequences of target genes via complementary pairing, leading to mRNA degradation or inhibition of target gene translation, thus exerting post-transcriptional control over target gene expression [8]. In recent years, numerous studies have underscored the crucial roles of miRNAs in skeletal muscle development. For example, *miR-27b-3p* regulates myoblast proliferation and differentiation by targeting myostatin gene [9]. *miR-21*, through its modulation of *TGFβ1* and the *PI3K/Akt/mTOR* signaling pathway, governs prenatal skeletal muscle development in pigs [10]. *miR-223-3p* promotes muscle regeneration through regulating inflammation [11]. *miR-322* exacerbates dexamethasone-induced muscle atrophy by targeting *IGF1R* and *INSR* [12].

*miR-103-3p*, a significant member of the miRNA family, has been reported to promote hepatic steatosis and exacerbate nonalcoholic fatty liver disease by targeting *ACOX1* [13]. It can also target the m6A methyltransferase *METTL14*, thereby inhibiting osteoblastic bone formation [14]. Furthermore, *miR-103-3p* regulates neural stem cell proliferation and differentiation by targeting *Ndel1* [15]. In our prior study, we observed that in dexamethasone-induced muscular atrophy models, the lncRNA *SYISL* binds to *miR-103-3p* and accelerates muscle atrophy [16], suggesting a potential role for *miR-103-3p* in mitigating muscle atrophy. However, the effects and mechanisms of *miR-103-3p* on muscle growth, myoblast proliferation, and differentiation remain unclear.

*BTG2*, a transcription factor which is a member of the BTG/Tob anti-proliferative protein family [17], could form mRNA deadenylation complexes with Ccr4-associated factor 1 (*CAF1*) and *CCR4*, thereby facilitating mRNA decay [18]. Its expression can be activated by *P53*, leading to the inhibition of the cell cycle process [19]. In addition, *BTG2* was also involved in many biological processes such as cell senescence [20], cell differentiation [21], oxidative damage [22] and DNA damage repair [23]. Then, what role does BTG2 play in the muscles? Studies have suggested that BTG2 may act as a regulator of MuSC aging and promote the senescence of muscle stem cells [23]. Yang et al. found that BTG2 may be the target gene of miR-222-3p, which could regulate the proliferation and differentiation of C2C12 myoblasts [24]. Ren et al. observed *BTG2* distribution in a model of 4 h skeletal muscle injury [25]. These findings collectively highlight the significant role of *BTG2* in muscle homeostasis and myogenic differentiation.

In our study, we observed high expression levels of *miR-103-3p* in mouse skeletal muscle. Through overexpression and inhibition experiments with *miR-103-3p* in C2C12 myoblasts, we found that *miR-103-3p* promotes the proliferation of C2C12 myoblasts while inhibiting their differentiation. The dual luciferase reporter assays further confirmed that *miR-103-3p* directly targets *BTG2* and regulates its expression, consequently influencing the proliferation and differentiation of C2C12 myoblasts. In conclusion, our study identifies *miR-103-3p* as a potential regulator of skeletal muscle growth and development.

## 2. Results

### 2.1. miR-103-3p Promotes the Proliferation of C2C12 Myoblasts

**Figure 1:**
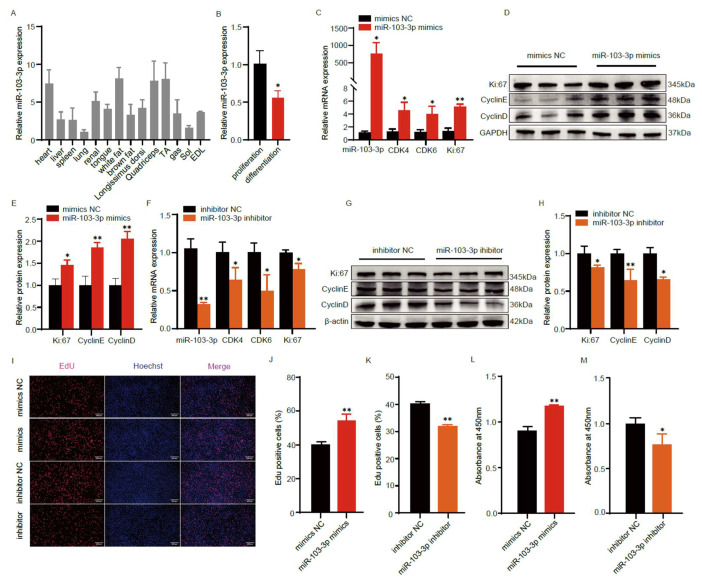
*miR-103-3p* promotes the proliferation of C2C12 myoblasts. (**A**) Relative expression level of *miR-103-3p* in 5-month-old mouse tissues. (**B**) The mRNA expression of *miR-103-3p* in the C2C12 myoblasts proliferation and differentiation. (**C**,**F**) The mRNA expression of *miR-10o3-3p*, *Ki67*, *CDK4* and *CDK6* after *miR-103-3p* mimics or inhibitor transfection were measured by RT-qPCR. (**D**,**E**,**G**,**H**) Protein expression of *Ki67*, *CyclinE* and *CyclinD* after *miR-103-3p* mimics or inhibitor transfection were measured by Western blot, and grayscale analysis were performed by Image J (2.6.1.0). (**I**–**K**) The proliferation of C2C12 myoblasts after *miR-103-3p* transfection was detected by EDU staining. S-phase myoblasts were stained with EdU (red) and nuclei with Hoechst (blue) and counted with Image J. The scale bar represents 200 μm. (**L**,**M**) CCK-8 analysis after treatment with *miR-103-3p* mimics and inhibitor during C2C12 myoblasts proliferation. Data are means ± SD (*n* = 3). * *p* < 0.05, ** *p* < 0.01.

To determine the expression pattern of *miR-103-3p* in skeletal muscle, we measured the expression level of *miR-103-3p* in the tissues of 5-month-old mice. The results showed that *miR-103-3p* was predominantly expressed in muscle and adipose tissue (Figure 1A). In previous studies, C2C12 myoblasts have been established as a valid model for studying skeletal muscle development [26]. Hence, we chose to conduct our research using C2C12 myoblasts. Our investigation revealed that *miR-103-3p* exhibited elevated expression levels during the initial phase of myoblast cell proliferation, with a subsequent decline in expression as the cells differentiated into myotubes (Figure 1B). To investigate the effects of *miR-103-3p* on the proliferation of C2C12 myoblasts, we transfected *miR-103-3p* mimics and inhibitor into C2C12 myoblasts. The real-time quantitative PCR (RT-qPCR) results showed that the overexpression of *miR-103-3p* significantly increased the expression level of proliferation-related genes *Ki67*, *CDK4* and *CDK6* (Figure 1C, *p* < 0.05). Additionally, the Western blot results showed that the overexpression of *miR-103-3p* significantly increased the expression level of proliferation-related genes *Ki67*, *Cyclin E* and *Cyclin D* (Figure 1D,E). Conversely, the inhibition of *miR-103-3p* led to a notable decrease in the expression levels of proliferation-related genes (Figure 1F–H). Furthermore, the EdU incorporation assay showed that the overexpression of *miR-103-3p* significantly promoted myoblast proliferation, and the knockdown of *miR-103-3p* significantly inhibited myoblast proliferation (Figure 1I–K). Similarly, CCK-8 experiment demonstrated that the overexpression of *miR-103-3p* could significantly promote the proliferation of myoblasts, while interference with *miR-103-3p* could significantly inhibit the proliferation of myoblasts. (Figure 1L,M). In summary, these results collectively demonstrate that *miR-103-3p* plays a pivotal role in promoting the proliferation of C2C12 myoblasts.

### 2.2. miR-103-3p Inhibited the Differentiation of C2C12 Myoblasts

**Figure 2:**
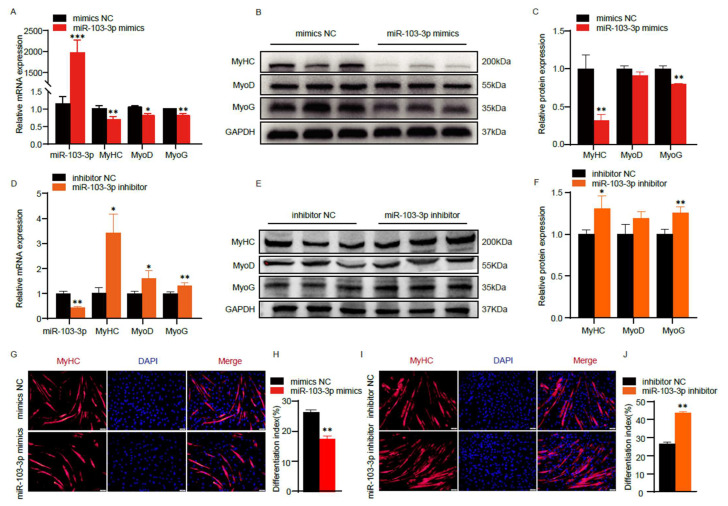
*miR-103-3p* inhibited the differentiation of C2C12 myoblasts. (**A**,**D**) The mRNA expression of *miR-103-3p*, *MyHC*, *MyoD* and *MyoG* after *miR-103-3p* mimics or inhibitor transfection were measured by RT-qPCR. (**B**,**C**,**E**,**F**) Differentiation marker genes protein expression of *MyHC*, *MyoD* and *MyoG* after *miR-103-3p* mimics or inhibitor transfection were measured by Western blot, and grayscale analysis were performed by ImageJ. (**G**–**J**) *MyHC* immunofluorescence staining and differentiation index after *miR-103-3p* overexpression and knockdown. The scale bar represents 50 μm. Data are means ± SD (*n* = 3). * *p* < 0.05, ** *p* < 0.01, *** *p* < 0.001.

Subsequently, we transfected mimics and inhibitor *miR-103-3p* into C2C12 cells to induce myoblast differentiation and assessed the effect of *miR-103-3p* on myoblast differentiation. The results from RT-qPCR and Western blot analyses clearly indicated that the overexpression of *miR-103-3p* led to a significant reduction in the expression levels of differentiation marker genes such as *MyHC*, *MyoD*, and *MyoG* (Figure 2A–C). Furthermore, immunofluorescence staining of *MyHC* confirmed that *miR-103-3p* overexpression inhibited myogenic differentiation (Figure 2G,H). In contrast, the knockdown of *miR-103-3p* resulted in a noteworthy increase in the expression of myoblast differentiation-related genes, including *MyoD*, *MyoG*, and *MyHC*, thereby promoting myoblast differentiation (Figure 2D–F,I,J). These findings collectively suggest that *miR-103-3p* possesses the capacity to impede the differentiation of C2C12 myoblasts.

### 2.3. miR-103-3p Directly Targeted BTG2

**Figure 3:**
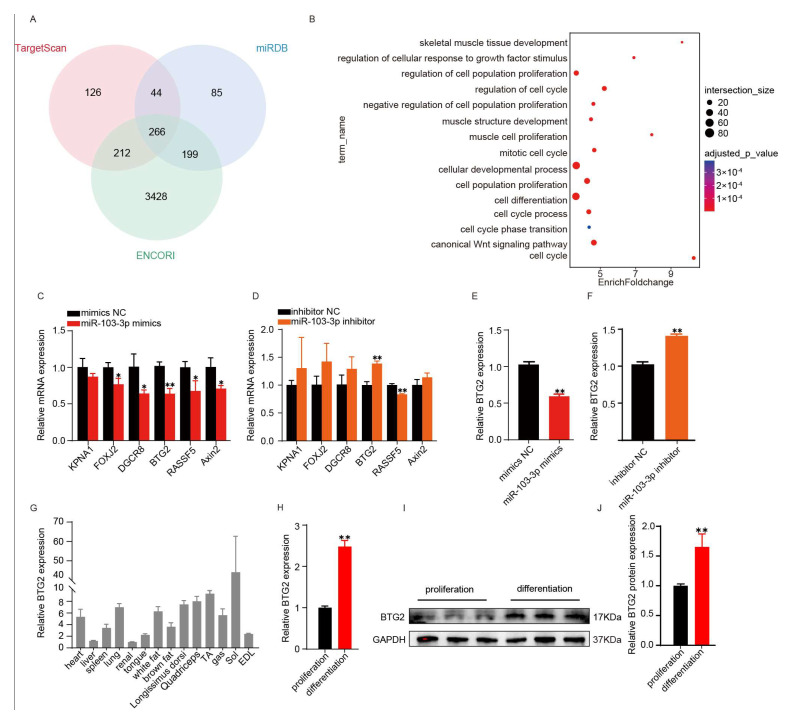

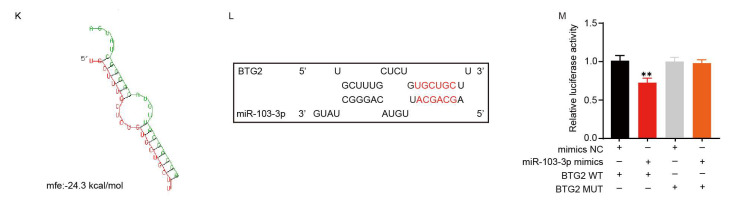
*miR-103-3p* directly targeted *BTG2*. (**A**) V The Venn diagram showed that miRDB, Targetsan and ENCORI predicted the target genes of *miR-103-3p*. (**B**) GO enrichment analysis revealed the enrichment pathway of *miR-103-3p* target genes. (**C**,**D**) Relative expression of *BTG2* mRNA at proliferation stage after treatment with *miR-103-3p* mimics and inhibitors. (**E**,**F**) Relative expression of *BTG2* mRNA at differentiation stage after treatment with *miR-103-3p* mimics and inhibitors. (**G**) Relative expression level of *BTG2* in 5-month-old mouse tissues. (**H**–**J**) mRNA and Western blotting analysis of *BTG2* protein expression in the myoblasts during proliferation and differentiation. (**K**,**L**) Schematic diagram and prediction of the binding site of *miR-103-3p* in the *BTG2* 3′UTR. The red font in figure (**L**) represents the binding site (**M**) Dual-luciferase reporter assays were performed after cotransfection of *miR-103-3p* mimics or mimics NC and psiCHECK2-BTG2-WT and psiCHECK2-BTG2-MUT vectors. The relative luciferase activity was presented as Renilla luciferase/firefly luciferase. Data are means ± SD (*n* = 3). * *p* < 0.05, ** *p* < 0.01.

As the small non-coding RNAs, miRNAs will regulate the expression of target genes mainly by binding to target gene mRNA. Therefore, we predicted the target genes of *miR-103-3p* with miRDB, targetscan and ENCORI online tool. This analysis yielded 266 potential binding target genes (Figure 3A). Furthermore, the Gene Ontology (GO) analysis revealed that these target genes were prominently associated with biological processes such as cell proliferation, cell development, and cell differentiation (Figure 3B). Among these target genes, we identified six that were particularly relevant to myoblast proliferation and differentiation: *KPNA1* [27], *FOXJ2* [28], *DGCR8* [29], *BTG2* [30], *RASSF5* [31] and *Axin2* [32]. To validate whether *miR-103-3p* could directly regulate the expression of these target genes, we conducted RT-qPCR experiments. The results showed that *miR-103-3p* could directly target and regulate the expression of *BTG2* in proliferating and differentiating myoblasts (Figure 3C–F). Subsequently, we examined the expression level of *BTG2* in the tissues of 5-month-old mice and found that *BTG2* was predominantly expressed in muscle (Figure 3G). We also measured the mRNA and protein expression level of BTG2 in C2C12 myoblasts during its 3-day differentiated myotubes and the results showed that *BTG2* was highly expressed during the proliferation and differentiation period (Figure 3H–J). Finally, dual-luciferase reporter assays provided compelling evidence that *miR-103-3p* mimics significantly inhibited the luciferase activity of the wild-type *BTG2* mRNA 3’ UTR reporter, while the dual fluorescence activity of the vector carrying the mutated *miR-103-3p* binding site remained largely unaffected (Figure 3K–M). These results demonstrated that *BTG2* could be a direct target gene of *miR-103-3p*.

### 2.4. BTG2 Inhibits the Proliferation and Promotes the Differentiation of C2C12 Myoblasts

**Figure 4:**
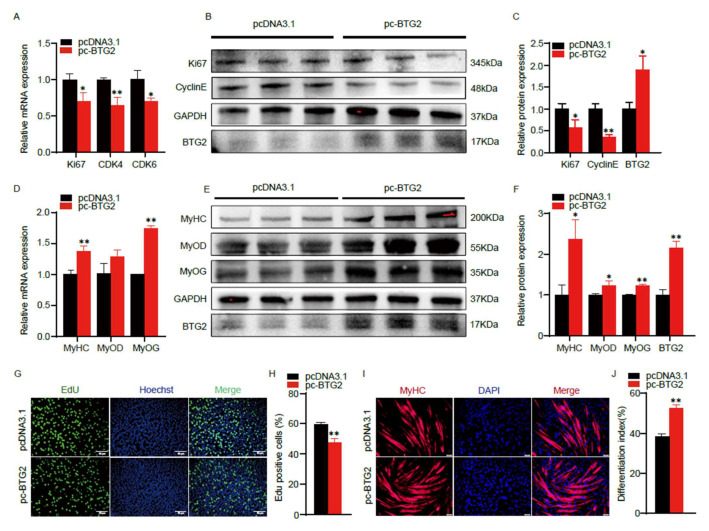
BTG2 inhibits the proliferation and promotes the differentiation of C2C12 myoblasts. (**A**,**D**) The mRNA expression of *Ki67*, *CDK4*, *CDK6*, *MyHC*, *MyoD* and *MyoG* after pc-BTG2 or pcDNA3.1 transfection were measured by RT-qPCR. (**B**,**C**,**E**,**F**) Protein expression of *BTG2*, *Ki67*, *CyclinE*, *CyclinD MyHC*, *MyoD* and *MyoG* after pc-BTG2 or pcDNA3.1 transfection were measured by Western blot, and grayscale analysis were performed by ImageJ. (**G**,**H**) The proliferation of C2C12 myoblasts after *BTG2* transfection wree detected by EDU staining. S-phase myoblasts were stained with EdU (green) and nuclei with Hoechst (blue) and counted with ImageJ. The scale bar represents 50 μm. (**I**,**J**) *MyHC* immunofluorescence staining and differentiation index after BTG2 overexpression. MyHC myotube (red) and nuclei with DAPI (blue). The scale bar represents 50 μm. Data are means ± SD (*n* = 3). * *p* < 0.05, ** *p* < 0.01.

To verify the role of *BTG2* in myogenesis, we overexpressed *BTG2* in C2C12 cells. The results demonstrated that *BTG2* significantly suppressed the mRNA expression of *Ki67*, *CDK4*, and *CDK6* (Figure 4A). In addition, the *BTG2* significantly down-regulated the protein expression of *Ki67*, *Cyclin E* and *Cyclin D* (Figure 4B,C). EdU staining revealed that *BTG2* significantly decreased the proportion of EdU-positive cells (Figure 4G), suggesting that *BTG2* inhibits the C2C12 myoblasts proliferation. Furthermore, overexpression of *BTG2* significantly increased the expression of the myogenic genes *MyHC*, *MyoD* and *MyoG* in mRNA (Figure 4D) and their protein level (Figure 4E,F). Immunofluorescence staining of *MyHC* showed that overexpression of *BTG2* significantly increased the number of myotubes (Figure 4H). In summary, these results provide strong evidence that *BTG2* has the capacity to inhibit the proliferation of C2C12 myoblasts and promote myogenic differentiation.

### 2.5. miR-103-3p Regulates Myogenesis by Targeting BTG2

**Figure 5:**
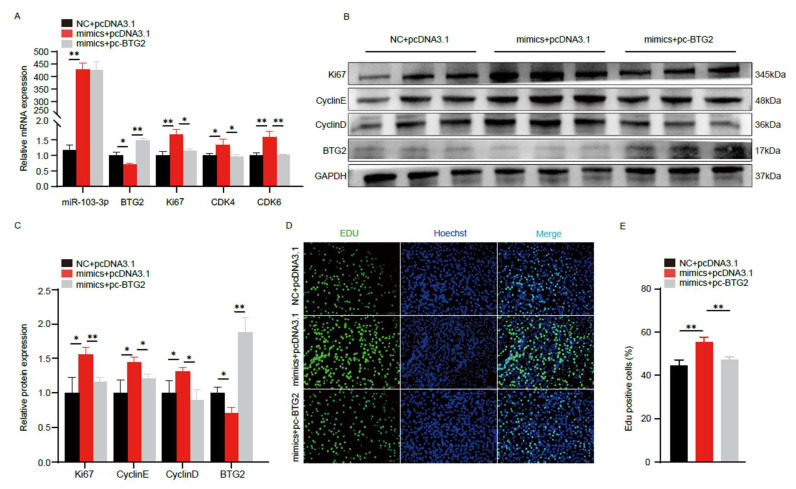
*miR-103-3p* promotes C2C12 myoblasts proliferation by targeting *BTG2*. (**A**) The mRNA expression levels of *miR-103-3p*, *BTG2*, *Ki67*, *CDK4* and *CDK6* after *miR-103-3p* and *BTG2* contransfection were measured by RT-qPCR. (**B**,**C**) The protein expression levels of the proliferation maker genes *Ki67*, *CyclinE* and *CyclinD* after contransfection of *miR-103-3p* and *BTG2*, and the grayscale analysis were performed by ImageJ. (**D**,**E**) The proliferation of C2C12 myoblasts after *miR-103-3p* and *BTG2* contransfection were detected by EdU staining. S-phase myoblasts were stained with EdU (green) and nuclei with Hoechst (blue) and counted with Image J. The scale bar represents 50 μm. Data are means ± SD (*n* = 3). * *p* < 0.05, ** *p* < 0.01.

To provide evidence that *miR-103-3p* promotes the proliferation of C2C12 myoblasts and inhibits myogenic differentiation primarily by targeting *BTG2*, we co-transfected *miR-103-3p* and *BTG2* overexpression vectors into C2C12 myoblasts. RT–qPCR results showed that the overexpression of *BTG2* could significantly offset the upregulation effect of the overexpression of *miR-103-3p* on the mRNA (Figure 5A) and protein level (Figure 5B,C) of myoblast proliferation genes. Additionally, EdU staining showed that overexpression of *BTG2* could effectively reduce higher ratio of EdU-positive cells resulting from the overexpression of *miR-103-3p* in C2C12 cells (Figure 5D,E). These findings strongly support the conclusion that *miR-103-3p* promotes the proliferation of C2C12 myoblasts by targeting *BTG2*.

**Figure 6:**
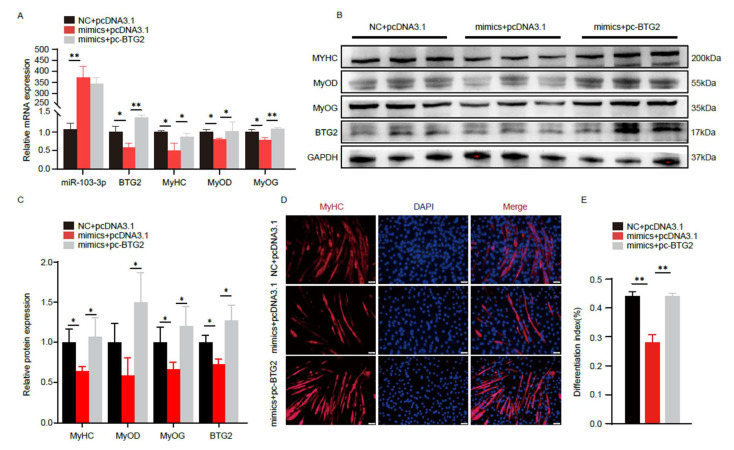
*miR-103-3p* inhibits C2C12 myoblasts differentiation by targeting *BTG2*. (**A**) The mRNA expression levels of *miR-103-3p*, *BTG2*, *MyHC*, *MyoD* and *MyoG* after *miR-103-3p* and BTG2 contransfection were measured by RT-qPCR. (**B**,**C**) The protein expression levels of the differentiation maker genes *MyHC*, *MyoD* and *MyoG* after contransfection of *miR-103-3p* and *BTG2*, and the grayscale analysis were performed by ImageJ. (**D**,**E**) *MyHC* immunofluorescence staining and differentiation index after *miR-103-3p* and *BTG2* contransfection. *MyHC* myotube (red) and nuclei with DAPI (blue). The scale bar represents 50 μm. Data are means ± SD (*n* = 3). * *p* < 0.05, ** *p* < 0.01.

Furthermore, we co-transfected *miR-103-3p* and *BTG2* overexpression vectors into C2C12 myoblasts to induce differentiation and then assessed the expression of related genes. The results revealed that the overexpression of *BTG2* mitigated the inhibitory effects of *miR-103-3p* on the expression of differentiation genes such as *MyHC*, *MyoD*, and *MyoG* (Figure 6A–C). Similarly, immunofluorescence staining of *MyHC* revealed that the overexpression of *BTG2* alleviated the inhibitory effect of *miR-103-3p* on myogenic differentiation (Figure 6D,E). In conclusion, these results strongly suggest that *miR-103-3p* inhibits C2C12 myogenic differentiation by targeting *BTG2*.

## 3. Discussion

Skeletal muscle growth and development represent intricate and finely regulated processes [33]. In this context, miRNAs have emerged as crucial players. For instance, *miR-33a* has been reported to hinder myoblast proliferation by targeting *IGF1*, follistatin, and cyclin D1 [34]. Similarly, *miR-743a-5p* has been shown to facilitate myoblast differentiation by targeting *Mob1b* in skeletal muscle development and regeneration [35]. Notably, an increasing number of miRNAs have been found to exhibit dual roles in myogenesis. For instance, *miR-100-5p* promotes proliferation while inhibiting differentiation of C2C12 myoblasts through the *Trib2/mTOR/S6K* signaling pathway [36]. Conversely, *miR-543* inhibits proliferation and promotes differentiation by targeting *KLF6* in C2C12 myoblasts [37]. In another example, *miR-21-5p* stimulates the proliferation and differentiation of skeletal muscle satellite cells by targeting *KLF3* in chickens [38]. Furthermore, *miR-668-3p* exerts inhibitory effects on myoblast proliferation and differentiation by targeting *Appl1* [39]. Our investigation into *miR-103-3p* has revealed its role in promoting proliferation while inhibiting differentiation in myoblasts, akin to the function of *miR-100-5p*. These findings underscore the pivotal role played by miRNAs in the intricate process of skeletal muscle development.

*miR-103-3p*, a highly conserved miRNA, can participate in various physiological regulatory processes. For instance, in gastric cancer, *miR-103* promotes proliferation and metastasis by targeting *KLF4* [40], while in endothelial maladaptation, it ameliorates the condition by targeting *lncWDR59*. However, this dual role implies that *miR-103-3p* may also hasten atherosclerosis [41]. We previously found that *SYISL* could act as a molecular sponge for *miR-103-3p*, weakening the inhibition of *miR-103-3p* on *MuRF1*, thus expediting muscle atrophy [16]. Therefore, *miR-103-3p* had an inhibitory effect on muscle atrophy. However, we found that *miR-103-3p* can promote myoblast proliferation and inhibit differentiation, a function that appears contradictory to its role in muscle atrophy. Similarly, *miR-23a* and *miR-186* have been reported to have similar functions. *miR-23a*, for instance, can suppress C2C12 myoblast differentiation through the downregulation of fast myosin heavy chain isoforms [42], yet it can simultaneously alleviate muscle atrophy caused by mice with chronic kidney disease (CKD) [43]. In C2C12 myoblasts, *miR-186* inhibits the muscle cell differentiation through myogenin regulation [44], while the expression level of *miR-186* was decreased in the in the vivo starvation induced muscular atrophy mouse model [45], which suggests that *miR-186* could alleviate muscular atrophy. This phenomenon can be understood as a difference in the regulatory network between normal muscle growth and muscle atrophy.

miRNAs exert their regulatory influence on cellular functions by binding to the 3’UTR sequences of various target genes [46]. Therefore, through bioinformatics analysis, we identified *BTG2*, a member of the antiproliferative (APRO) gene family [47], as a potential target gene of *miR-103-3p*. This selection allowed us to delve into the molecular mechanism through which *miR-103-3p* regulates the proliferation and differentiation of C2C12 myoblast cells. *BTG2* has been implicated in a wide range of physiological and pathological processes, including cell proliferation [48], differentiation [21], and apoptosis [49]. Furthermore, *BTG2* has been found to be involved in skeletal muscle growth and development. Feng et al. reported that *BTG2* may inhibit the proliferation of primary muscle fibers and play a role in the differentiation process of C2C12 myoblasts [50]. Additionally, studies have shown that the expression of *BTG2* in Ziwuling black goats with low meat yield was higher than that in Liaoning cashmere goats with high meat yield [51]. *miR-222-3p* has also been demonstrated to regulate the proliferation and differentiation of C2C12 myoblasts by targeting *BTG2* [38].

In summary, our results indicate that *BTG2* possesses the capacity to inhibit proliferation and promote differentiation of C2C12 myoblasts. Thus, *miR-103-3p*, which plays a significant role in skeletal muscle growth and development, can promote proliferation and inhibit differentiation of C2C12 myoblasts by targeting *BTG2*. However, the specific pathway mechanism underlying the regulatory effects of *BTG2*, bound to *miR-103-3p*, on myoblast proliferation and differentiation, warrants further investigation.

## 4. Materials and Methods

### 4.1. Cell Culture

C2C12 myoblasts and HEK293T cells were cultured in a growth medium composed of high-glucose DMEM (DMEM Hyclone, Logan, UT, USA) supplemented with 10% fetal bovine serum (Gibco, Grand Island, NY, USA) in a cell incubator maintained at 37 °C with 5% CO_2_ in a humidified environment. Differentiation of C2C12 cells was induced by switching to DMEM containing 2% horse serum (Gibco, Grand Island, NY, USA) when cell fusion reached 80%. Three independent repetitions of the entire experiment, along with three repetitions within a single experiment.

### 4.2. RNA Oligonucleotides and Cell Transfection

To explore the effects of *miR-103-3p* and its target gene on C2C12 myoblasts, we synthesized the *miR-103-3p* inhibitor, an inhibitor negative control (inhibitor NC), *miR-103-3p* mimic, negative control (mimic NC or siRNA NC) from GenePharma (GenePharma, Shanghai, China). we co-transfected C2C12 myoblasts with 50 nM miR-103-3 or mimics NC and 3 μg BTG2 plasmid or 3 μg pcDNA3.1 using 4 μL Lipofectamine 2000 (Invitrogen, Carlsbad, CA, USA) in each well of a 6-well plate. For the proliferation experiments, C2C12 myoblast transfection was performed when cell density reached 40%. After 6 h of transfection, we changed the medium to a growth medium. After 24 h, the samples were received. For the differentiation experiments, transfection was performed when the cell density reached 80%. After 6 h of transfection, the medium was replaced with a differentiation medium. All RNA oligonucleotides are listed in Table 1.

### 4.3. RNA Extraction and Real-Time Quantitative PCR (RT-qPCR)

Total RNA was extracted from C2C12 myoblast using TRIzol reagent (Takara Bio, Otsu, Japan) according to the manufacturer’s instructions. RNA was reverse-transcribed to cDNA using the PrimeScript RT Reagent Kit (Takara Bio, Otsu, Japan). Real-time quantitative polymerase chain reaction (RT-qPCR) was performed using SYBR premixed Ex Taq kit (Vazyme Biotech, Nanjing, China). We used the 2^−ΔΔCt^ method to quantify the target genes relative to mRNA expression level. mRNA expression was normalized relative to GAPDH, and U6 was used to normalize *miR-103-3p* expression. The sequence information of primers is listed in Table 2.

### 4.4. Western Blot

Proteins were extracted from cells using radioimmunoprecipitation assay (RIPA) buffer with 1% (*v*/*v*) reverse transcription kits (Cwbio, Taizhou, Zhejiang, China). The total protein sample was separated in the SDS-polyacrylamide gel. Then, it was transferred into a PVDF membrane (Millipore, Bedford, MA, USA). Next, the membrane was blocked in 5% defatted milk for 2 h. The primary antibody 4 was incubated overnight. The antibodies used included *Ki67* (1:1000; Abcam, Cambridge, UK), *CyclinD* (1:1000; ProteinTech, Wuhan, China), *CyclinE* (1:1000; ProteinTech, Wuhan, China), *MyHC* (1:1000; ProteinTech, Wuhan, China), *MyoD* (1:1000; ProteinTech, Wuhan, China), *MyoG* (1:1000; ProteinTech, Wuhan, China), and *GAPDH* (1:2000; ProteinTech, Wuhan, China). After incubation, the membrane was washed three times with TBST solution, and secondary antibodies (Goat Anti-Mouse IgG, Boster, BA1038; Goat Anti-Rabbit IgG, Boster, BA1039; Boster Biological Technology, Pleasanton, CA, USA) were added. Finally, Western blots were exposed to the Bio-Rad imaging system. All protein levels were normalized to that of the glyceraldehyde-3-phosphate dehydrogenase (*GAPDH*), and densitometric quantification of the Western blotting bands was performed using ImageJ (2.6.1.0) software.

### 4.5. Immunofluorescence Staining

The differentiated C2C12 myoblasts were fixed with 4% paraformaldehyde at room temperature for 30 min and then permeated with 0.5% Triton-100 for 30 min. Cells washed with PBS were blocked with 5% bovine serum albumin (BSA) (Biofroxx, Berlin, Germany) at room temperature for 1 h. Subsequently, the cells were incubated overnight with primary antibodies against *MyHC* (1:100; R&D, Minneapolis, MN, USA) at 4 °C. Following three washes with PBS, the cells were incubated with the appropriate fluorescent secondary antibody at room temperature for 1 h. Finally, the nucleus was stained with DAPI for 10 min. Then, the cells were photographed and counted under a fluorescence microscope.

### 4.6. 5-Ethynyl-20-Deoxyuridine (EdU) Assay

C2C12 myoblasts were seeded into 96-well cell culture plates, and transfections were carried out once the cell density reached 30–40%. After 24 h of transfection, the cells were processed following the instructions of the Cell-LightTM EdU Apollo567 In Vitro Kit (RiboBio, Guangzhou, China). Subsequently, the cells were captured under a fluorescence microscope.

### 4.7. CCK-8 Assay

C2C12 myoblasts were plated in 96-well cell culture plates, with each well receiving 2 × 10^3^ cells. Transfection was conducted when the cell density reached 30–40%. After 24 h, 10 μL of Cell-Counting Kit-8 (CCK-8) reagents (Solarbio, Beijing, China) were added to the cells for a 2 h incubation period. Subsequently, the absorbance of the cells at 450 nm was measured using an enzyme-labeled instrument, and the data were subjected to statistical analysis.

### 4.8. Dual-Luciferase Reporter Assay

The *BTG2* 3’-UTR was custom-synthesized by General Biology Systems Ltd. (Chuzhou, Anhui, China). Human embryonic kidney 293T cells (obtained from the Stem Cell Bank of the Chinese Academy of Sciences) were seeded into 48-well culture plates at a density of 8000 cells per well. Subsequently, psiCHECK2-BTG2-WT and psiCHECK2-BTG2-MUT plasmids were co-transfected with either 50 nM of *miR-103-3p* mimics or mimics nc when the cells reached a confluence of 70%. After 48 h of transfection, we measured the relative luciferase activities of Renilla compared to those of firefly using a Dual-Luciferase reporter assay system (Promega, E1910; Madison, WI, USA), following the manufacturer’s protocol.

### 4.9. Bioinformation Analysis

The potential target genes of *miR-103-3p* were predicted using multiple platforms, including TargetScan 7.1 for the mouse (http://www.targetscan.org, accessed on 23 April 2023), miRDB (http://www.miRdb.org/miRDB/, accessed on 23 April 2023), and ENCORI (https://rnasysu.com/encori/index.php, accessed on 23 April 2023). Subsequently, these target genes were subjected to Gene Ontology (GO) enrichment analysis (https://biit.cs.ut.ee/gprofiler/convert, accessed on 23 April 2023). The significance threshold for enrichment was established at a corrected *p*-value of <0.05.

### 4.10. Statistical Analysis

All statistical analyses were conducted using GraphPad Prism 8.02. The data are presented as the mean ± standard deviation. Significance levels were determined using Student’s *t*-test or one-way and two-way analysis as appropriate (*, *p* < 0.05; **, *p* < 0.01), indicating the significance of differences between the groups.

## 5. Conclusions

**Figure 7:**
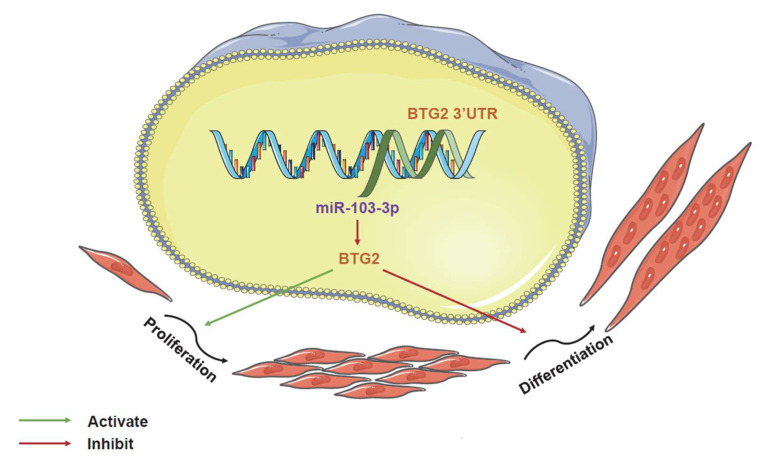
*miR-103-3p* promotes proliferation and inhibits differentiation of C2C12 myoblasts by targeting *BTG2*.

In summary, our findings suggest that *miR-103-3p* enhances the proliferation of C2C12 myoblasts while simultaneously inhibiting their differentiation by targeting *BTG2*, as illustrated in Figure 7.

## Figures and Tables

**Table 1:** RNA oligonucleotides used in this study.

Gene	Primer Sequences	
	Forward Primer	Reverse Primer
*GAPDH*	AGGTCGGTGTGAACGGATTTG	TGTAGACCATGTAGTTGAGGTCA
*Ki67*	ATCATTGACCGCTCCTTTAGGT	GCTCGCCTTGATGGTTCCT
*CDK4*	AGTTTCTAAGCGGCCTGGAT	AACTTCAGGAGCTCGGTACC
*CDK6*	GGCGTACCCACAGAAACCATA	AGGTAAGGGCCATCTGAAAACT
*MyHC*	ACGATGGACGTAAGGGAGTGCAGAT	TGTCGTACTTGGGCGGGTTC
*MyOD*	CGAGCACTACAGTGGCGACTCAGAT	GCTCCACTATGCTGGACAGGCAGT
*MyOG*	CCATCCAGTACATTGAGCGCCTACA	ACGATGGACGTAAGGGAGTGCAGAT
*miR-103-3p*	AACACGCAGCAGCATTGTAC	GTCGTATCCAGTGCAGGGT
*U6*	GTGCTCGCTTCGGCAGCACATAT	AAAATATGGAACGCTTCACGAA
*BTG2*	GGTTGGAGAAAATTGGGAAAC	GCTTCTAAGAAGCCCTCATC

**Table 2:** Primer information for miRNA and mRNA quantitative reverse transcription.

Name	Sequence (5′ to 3′)
*miR-103-3p* mimic	AGCAGCAUUGUACAGGGCUAUGA
	AUAGCCCUGUACAAUGCUGCUUU
mimics NC	UUCUCCGAACGUGUCACGUTT
	ACGUGACACGUUCGGAGAATT
*miR-103-3p* inhibitor	UCAUAGCCCUGUACAAUGCUGCU
inhibitor NC	CAGUACUUUUGUGUAGUACAA

## Data Availability

Data from this study are included in the article.
